# Repositioning of the Antihyperlipidemic Drug Fenofibrate for the Management of *Aeromonas* Infections

**DOI:** 10.3390/microorganisms12030465

**Published:** 2024-02-25

**Authors:** Roberto M. Guerra, Maria José Figueras, Isabel Pujol-Bajador, Ana Fernández-Bravo

**Affiliations:** 1 Unit of Microbiology, Department of Basic Health Sciences, Faculty of Medicine and Health Sciences, Institut d’Investigació Sanitària Pere Virgili (IISPV), University Rovira i Virgili, 43201 Reus, Spain; juanroberto.monllor@urv.cat (R.M.G.); mariajose.figueras@urv.cat (M.J.F.); 2 Microbiology Laboratory, University Hospital Sant Joan de Reus, Salut Sant Joan de Reus-Baix Camp, 43204 Reus, Spain

**Keywords:** fenofibrate, drug repositioning, *Aeromonas*, infection, immune response, antimicrobial resistance

## Abstract

Fenofibrate is a fibric acid derivative used as an antihyperlipidemic drug in humans. Its active metabolite, fenofibric acid, acts as an agonist to the peroxisome proliferator-activated receptor alpha (PPAR-α), a transcription factor involved in different metabolic pathways. Some studies have reported the potential protective role of this drug in cell lines and in vivo models against bacterial and viral infections. The aim of this study was to assess the in vitro effect of fenofibrate in the macrophage cell line J744A.1 against infections produced by *Aeromonas*, a pathogen for humans whose resistance to antibiotics has increased in recent decades. Macrophages were infected at MOI 10 with four strains of *Aeromonas caviae* and *Aeromonas hydrophila* isolated from human clinical samples and subsequently treated with fenofibrate. It was observed that fenofibrate-treated macrophages showed lower levels of cytotoxicity and intracellular bacteria compared to non-treated macrophages. In addition, the viability of treated macrophages was dependent on the dose of fenofibrate used. Furthermore, transcriptional analysis by RT-qPCR revealed significant differences in the expression of the *PPAR-α* gene and immune-related genes *TNF-α*, *CCL3*, and *BAX* in fenofibrate-treated macrophages compared to the macrophages without treatment. This study provides evidence that fenofibrate offered some protection in vitro in macrophages against *Aeromonas* infection. However, further studies are needed with other bacteria to determine its potential antibacterial effect and the route by which this protection is achieved.

## 1. Introduction

Antimicrobial resistance (AMR) is currently one of the most concerning global health threats [1]. This phenomenon arises when microorganisms experience changes in their susceptibility to antimicrobials over time and become unresponsive to them, making infections more difficult to treat, enhancing the potential for spreading the disease they produce, and increasing associated morbidity and mortality [2,3]. The problem of AMR is particularly alarming in bacteria. Some examples of bacteria that represent a particular menace in healthcare settings due to their multiresistant character include Methicillin-resistant *Staphylococcus aureus* (MRSA), extended-spectrum β-lactamase-producing and carbapenemase-producing enterobacteria, *Acinetobacter* or *Pseudomonas* [4,5,6]. These bacteria can cause severe and often deadly infections, such as bloodstream infections and pneumonia, with antibiotic therapy generally not effective. Antibiotic resistance leads to an estimated 700,000 deaths each year globally, and some evaluations project this number to increase to 10 million by the year 2050 if steps are not taken to develop new effective compounds with antimicrobial activity [7]. One of the main factors that has greatly influenced the development of multidrug-resistant (MDR) bacteria is the overuse and inappropriate management of antibiotics for prophylactic and therapeutic purposes, accelerating the natural process of acquired AMR [8]. Another consideration that is increasing the severity of this problem is the gradual slowdown that the pharmaceutical industry has experienced in the discovery and development of new antibiotics and the production of their semi-synthetic derivatives during the last decades [9]. In this sense, the development of de novo antibiotics requires long research time and significant economic investment, and in most cases, it faces low profitability and success rates [10]. Considering all the stages necessary for its approval by a safety medical agency, it takes no less than ten years to put a drug on the market [10,11]. Additionally, some estimations report that only between one and two drugs from 10,000 compounds reach Federal Drug Administration (FDA) approval [11]. Meanwhile, the application of known drugs or compounds to new indications, a strategy generally referred to as drug repositioning or drug repurposing, has been proposed as a viable solution to address this problem in the short term [12]. The main advantage of these drugs is that they have already been approved for their original application, evading several steps of this long and uncertain process. Several studies have explored the potential repositioning of some drugs already used for their original indications for being used in the treatment of bacterial infections [13,14]. These kinds of drugs may have antimicrobial activity, interfere with bacterial virulence mechanisms, or improve the host response against the harmful effects caused by bacterial invasion and proliferation [13,14]. In this sense, different drug groups have been found effective against bacterial infections, such as some Non-Steroidal Anti-Inflammatory Drugs (NSAIDs), antidepressants and antipsychotics, or statins [13,14]. In the same way, the FDA-approved drug fenofibrate has been considered a promising target for repositioning against bacterial infections based on previous in vitro and in vivo studies [15,16,17,18]. Fenofibrate belongs to the family of fibrates, derived from fibric acid. It is used as an anti-hyperlipidemic drug since its metabolite fenofibric acid acts as an agonist to the peroxisome proliferator-activated receptor alpha (PPAR-α), a transcription factor involved in lipid metabolism regulation among other cell pathways with reported anti-inflammatory, antiangiogenic, antiapoptotic, and antioxidant effects [19,20,21,22]. Indeed, the fenofibrate-induced PPAR-α activation has been shown to be an effective way to improve immune response both in in vitro and in vivo models facing bacterial challenges [16,17,18]. In this study, we focused on studying the potential protective role of fenofibrate in the murine macrophage cell line J774A.1 against infections produced by clinical strains of *Aeromonas*, a bacterial genus ubiquitous in aquatic ecosystems for which the mechanisms of virulence have been extensively studied [23,24,25]. Several *Aeromonas* species are considered emergent opportunistic pathogens for humans, with the most common diseases associated with them being gastroenteritis, septicemia, and soft-tissue infections [23,25], producing, in some cases, severe necrotizing fasciitis [26,27,28]. Antibiotic treatment for *Aeromonas* infections is, in some cases, diminished due to the apparition of MDR strains. The aim of this study was to analyze the potential activity of fenofibrate against different clinical *Aeromonas* strains in an experimental infection model using the murine macrophage cell line J774A.1.

## 2. Materials and Methods

### 2.1. Bacterial Strains and Culture Conditions

Four bacterial isolates from stool cultures of patients with diarrhea (1127C, 1172C, 59798, and 111851) were preliminary identified as *Aeromonas* sp. based on matrix-assisted laser desorption ionization time-of-flight (MALDI-TOF). The in vitro antimicrobial susceptibility profiles were determined with Vitek 2 (bioMérieux, Marcy-l’Étoile, France). Their categorization as having susceptibility, or intermediate or full antibiotic resistance, was based on the breakpoints established by the Clinical and Laboratory Standards Institute for the tested antibiotics [29], revealing different susceptibility profiles (Table 1). A molecular identification was performed by sequencing the *rpoD* housekeeping gene. Genomic DNA was extracted from pure cultures grown in Difco^TM^ Tryptic Soy Agar (TSA, Becton Dickinson and Company, Sparks, MD, USA) using the InstaGene^TM^ DNA purification matrix (Bio-Rad Laboratories, Inc., Hercules, CA, USA) and following the instructions of the manufacturer. PCR of the *rpoD* gene was performed with the primers and conditions described by Soler et al. [30] (Table 2). Amplicons with an expected size of 820 base pairs (bp) [30] were verified in a 1% agarose gel electrophoresis containing the RedSafe^TM^ nucleic acid staining solution (iNtRON Biotechnology, Seongnam, Republic of Korea) and visualized using a Molecular Imager^®^ Gel Doc^TM^ XRT and the Image Lab^TM^ software version 5.0, both from Bio-Rad. The amplicons were sequenced and then aligned with the *rpoD* sequences of the type strains of *Aeromonas* species with the ClustalW algorithm [31] in MEGA v7.0 [32]. The phylogenetic analysis was performed using the neighbor-joining (NJ) algorithm in MEGA v7.0. Bacterial strains were stored at −80 °C in Difco^TM^ Tryptic Soy Broth (TSB, Becton Dickinson and Company) with 15% of glycerol (Panreac Applichem ITW Reagents, Monza, Italy). Bacteria were grown in TSA at 30 °C for 24 h before experiments.

### 2.2. Virulence Gene Detection

The presence of genes associated with *Aeromonas* virulence, including aerolysin (*aerA*), hemolysin (*hlyA*), cytotoxic enterotoxin (*act*), cytotonic enterotoxins (*ast* and *alt*), the flagellin A (*flaA*) gene, and Type III secretion system genes (*ascF*, *ascV*, *aexT*) was assessed by PCR using specific primers (Table 2) and PCR conditions described previously [33].

### 2.3. Fenofibrate In Vitro Antimicrobial Activity

The potential antimicrobial effect of fenofibrate on the *Aeromonas* strains used in this study was evaluated with a broth microdilution assay [34] followed by monitoring bacterial growth through optical density reading [35]. Bacterial suspensions of all *Aeromonas* strains to be tested were prepared in Phosphate Buffer Saline (PBS) from overnight cultures in TSA and adjusted to the standard 0.5 McFarland turbidity. These suspensions were diluted in TSB in a ratio of 1:100, and 100 µL were inoculated in a 96-well microplate. A stock of fenofibrate (purchased from Sigma-Aldrich, Saint Louis, MO, USA in powder form) was prepared in TSB, and 100 µL each of different dilutions was mixed with bacteria in the microplate to reach final concentrations of 10, 33, and 50 µM. Then, bacterial growth curves were determined by measuring the optical density of 600 nm (OD_600_ nm) for 18 h at 30 °C in an Agilent BioTek 800 TS microplate reader (Agilent Technologies, Inc., Santa Clara, CA, USA). The assay was conducted in quadruplicates, including negative controls consisting of bacterial suspension of each strain in TSB without fenofibrate and positive controls of antimicrobial activity using chloramphenicol (Sigma-Aldrich) in TSB at a concentration of 10 µM.

**Table 2:** Primers used in this study.

Primer	Sequence (5′–3′)	Target	Reference
rpoD 70Fs1	GTCAATTCCGCCTGATGC	*rpoD*	[30]
rpoD 70Rs1	ATCATCTCGCGCATGTTGT		
ASCF-G-fwd	ATGAGGTCATCT GCT CGC GC	*ascF-G*	[36]
ASCF-G-rev	GGAGCACAACCATGGCTGAT		
ASCV-fwd	ATGGACGGCGCCATGAAGTT	*ascV*	[36]
ASCV-rev	TATTCGCCTTCACCCATCCC		
aerA forward	GC(A/T)GA(A/G)CCC(A/G)TCTATCC(A/T)G	*aerA*	[37]
aerA reverse	TTTCTCCGGTAACAGGATTG		
hylA forward	GGCCGGTGGCCCGAAGATACGGG	*hlyA*	[37]
hylA reverse	GGCGGCGCCGGACGAGACGGG		
act forward	GAGAAGGTGACCACCAAGAAGA	*act*	[37]
act reverse	AACTGACATCGGCCTTGAACTC		
ast-F	ATCGTCAGCGACAGCTCTT	*ast*	[37]
ast-R	CTCATCCCTTGGCTTGTTGT		
flaA forward	TCCAACCGTYTGACCTC	*flaA*	[38]
flaA reverse	GMYTGGTTGCGRATGGT		
alt-F	AAAGCGTCTGACAGCGAAGT	*alt*	[39]
alt-R	AGCGCATAGGCGTTCTCTT		
aexT forward	GGCGCTTGGGCTCTACAC	*aexT*	[40]
aexT reverse	GAGCCCGCGCATCTTCAG		
GAPDH forward	CATGAGAAGTATGACAACAGCCT	*GAPDH*	[41]
GAPDH reverse	AGTCCTTCCACGATACCAAAGT		
PPAR-α.1	GTGGCTGCTATAATTTGCTGTG	*PPAR-α*	[42]
PPAR-α.2	GAAGGTGTCATCTGGATGGGT		
TNF-α forward	GAGGCCAAGCCCTGGTATG	*TNF-α*	[41]
TNF-α reverse	CGGGCCGATTGATCTCAGC		
CCL3 forward	AGTTCTCTGCATCACTTGCTG	*CCL3*	[41]
CCL3 reverse	CGGCTTCGCTTGGTTAGGAA		
BAX forward	CCCGAGAGGTCTTTTTCCGAG	*BAX*	[41]
BAX reverse	CCAGCCCATGATGGTTCTGAT		

### 2.4. Macrophage Cell Line, Reagents, and Growth Conditions

The cell line J774A.1 from mouse BALB/C monocytes/macrophages was purchased from the American Type Culture Collection (ATCC) in frozen vials. For cell cultures, Dulbecco’s Modified Eagle’s Medium (DMEM), fetal bovine serum (FBS), and penicillin-streptomycin stock (P/S) were purchased from PAA Laboratories GmbH, Munich, Germany. Cells J774A.1 were maintained in adhesion in DMEM (pH = 8) supplemented with 10% FBS plus 1% P/S solution at 37 °C and 5% CO_2_. Before infection, cells were seeded in tissue culture plates containing serum and antibiotic-free DMEM for 24 h to form confluent monolayers, as described in Guerra et al. [43]. For in vitro infection experiments in macrophages, fenofibrate was prepared in DMEM solution at concentrations of 10, 33, and 50 µM.

### 2.5. Determination of Intracellular Bacterial Survival in Macrophages following Fenofibrate Treatment

To assess the effect of fenofibrate on the survival of *Aeromonas* in macrophages, a quantitative determination of intracellular bacteria after incubation with fenofibrate was determined by the gentamicin exclusion assay [27,28]. Macrophages were seeded in 6-well plates for 24 h to obtain confluent monolayers and then infected with each of the four *Aeromonas* strains at a multiplicity of infection (MOI) of 10, as described previously [43]. Co-cultures of bacteria–macrophages were incubated at 37 °C and 5% CO_2_ for 1 h. Then, the medium was replaced with fresh DMEM with gentamicin (50 μg/mL) in all the wells for 45 min to kill extracellular bacteria not internalized in macrophages and considering at this point the starting intracellular bacterial load (t0). After that, the medium was replaced with fresh DMEM with fenofibrate 33 µM in the wells of fenofibrate-treated macrophages, and only with DMEM in wells of macrophages without treatment. Plates were incubated at 37 °C and 5% CO_2_ for 16 h, considering this the end point of incubation (t16). Bacterial loads present inside macrophages at t0 and t16 were determined by serial dilution in PBS, and plating in TSA [28,43]. The CFU/mL were calculated after incubation of TSA plates at 30 °C for 24 h. The percentages of intracellular bacterial survival were calculated with the number of CFU/mL at t16 in relation to CFU/mL at t0. Resting cells, i.e., non-infected macrophages in DMEM, were used as controls.

### 2.6. Quantification of Cell Damage in Macrophages following Infection and Fenofibrate Treatment

The macrophages were seeded in 6-well plates for 24 h to obtain confluent monolayers and then infected with *Aeromonas* strains at MOI 10. The co-cultures of bacteria–macrophages were then incubated at 37 °C and 5% CO_2_ for 1 h (t0). Subsequently, the medium was replaced with fresh DMEM with fenofibrate 33 µM in the wells of fenofibrate-treated macrophages, and only with DMEM in wells of macrophages without treatment. The plates were then incubated at 37 °C and 5% CO_2_ for 16 h (t16). Cell damage in macrophages at t0 and t16 was assessed by quantifying the released lactate dehydrogenase enzyme (LDH) into the culture supernatants [33,43], using the Cytotox 96 Non-Radioactive Cytotoxicity Assay^®^ (Promega, Madison, WI, USA), following the instructions of the manufacturer. A standard curve was generated with recombinant bovine LDH, and sample values were extrapolated from there. Percentages of reduction in cell damage of fenofibrate-treated macrophages at t16 were calculated with respect to macrophages without treatment at t16. Resting cells, i.e., non-infected macrophages in DMEM, were used as controls.

### 2.7. Screening for Fenofibrate Dose-Response Effect on Macrophage Viability following Infection with Highly Virulent and Multiresistant 1127C Strain

The MTT Cell Proliferation Assay 30-1010K™ (ATCC) was used as a measure of macrophage viability after infection with *Aeromonas caviae* 1127C strain and fenofibrate treatment at different doses. Macrophages were seeded in 96-well microtiter plates with a concentration of 2 × 10^4^ cells/well to form confluent monolayers in a total volume of 100 µL/well [17]. Then, macrophages were infected with 1127C strain at MOI 10 and subsequently incubated with gentamicin 50 µg/mL for 45 min. Later, the medium was replaced with fresh DMEM with fenofibrate at concentrations of 10, 33, and 50 µM, and plates were incubated for 12 h at 37 °C and 5% CO_2_. Additionally, macrophages were incubated with the same concentrations of fenofibrate in the absence of infection to assess their viability and possible cytotoxic effects on the cells. After incubation, the MTT reagent was added to the wells (10 µL/well), and microplates were incubated at 37 °C and 5% CO_2_ for 2 h. At that point, 100 µL of the detergent reagent was added following incubation in darkness at room temperature for 2 h. Finally, absorbance was measured at 570 nm in a Spectramax M5e microplate reader (Molecular Devices, Sunnyvale, CA, USA). Viability percentages were calculated with respect to resting cells i.e., non-infected macrophages incubated with DMEM, for which a value of 100% of viability was established.

### 2.8. Analysis of the Effect of Fenofibrate in the Expression of PPAR-α and Genes Related to the Innate Immune Response

The effect of fenofibrate on transcript levels of the *PPAR-α* gene and innate immune response-related genes *TNF-α*, *CCL3*, and *BAX* in macrophages against infection with strain 1127C was assessed with RT-qPCR. Macrophage seeding for obtaining confluent monolayers and infection with 1127C bacteria at MOI 10 was performed in 6-well plates as described previously. Then, one group of infected macrophages was treated with fenofibrate at a concentration of 33 µM, while another group was not treated. After that, plates were incubated at 37 °C and 5% CO_2_ for 16 h, isolating RNA at 4 and 16 h from different wells. Briefly, macrophages were washed twice with PBS, and the RNA was isolated from the samples using the High Pure RNA Isolation Kit (Roche Diagnostics GmbH, Mannheim, Germany). The quality and integrity of RNA were evaluated using NanoDrop^TM^ 2000 (Thermo Scientific, Wilmington, DE, USA), using the 260/280 and 260/230 nm ratios as quality parameters. Then, transcription of cDNA from total RNA was performed with the iScript cDNA Synthesis Kit (Bio-Rad Laboratories). A real-time PCR was performed with cDNA by using the Power SYBR^®^ green PCR Mastermix (Applied Biosystems, Life Technologies, Glasgow, UK) and a StepOnePlus™ Real-Time PCR System (Applied Biosystems) with specific primers for innate immune genes [41] and *PPAR-α* [42] (Table 2). Threshold cycle (CT) values were used to establish the relative expression levels of the studied genes, using the glyceraldehyde-3-phosphate dehydrogenase (*GAPDH*) gene as a housekeeping gene of reference [33]. Finally, the relative gene expression was determined using the delta-delta Ct (2^−ΔΔCt^) method that relays the signal from the real-time PCR. Results were expressed as fold changes in relation to the resting cells, i.e., non-infected macrophages in DMEM.

### 2.9. Testing Fenofibrate as a Therapeutic in Model of Pseudomonas aeruginosa Infection

The macrophages were seeded in 6-well plates for 24 h to obtain confluent monolayers and then infected with *Pseudomonas aeruginosa* ATCC 10145 at MOI 5. The co-cultures of bacteria–macrophages were then incubated at 37 °C and 5% CO_2_ for 1 h (t0). Subsequently, the treatment was performed with fenofibrate at 33 µM and the plates were then incubated at 37 °C and 5% CO_2_ for 16 h (t16). Cell damage in macrophages at t0 and t16 was determined by quantifying the LDH using the Cytotox 96 Non-Radioactive Cytotoxicity Assay^®^ (Promega), as described previously for *Aeromonas.* The percentage of intracellular bacterial survival was calculated with the number of CFU/mL at t16 in relation to CFU/mL at t0.

### 2.10. Statistical Analysis

All in vitro infection experiments were performed in triplicate. Significant differences between different conditions were determined using Student’s two-tailed *t*-test with Welch’s correction and calculated on Graph Pad Prism 6.0 (GraphPad Prism Software Inc., San Diego, CA, USA). Data were considered statistically significant at *p*-values < 0.05 (*), <0.01 (**), and <0.001 (***).

## 3. Results

### 3.1. Molecular Identification of Aeromonas Strains Based on rpoD Housekeeping Gene

**Figure 1:**
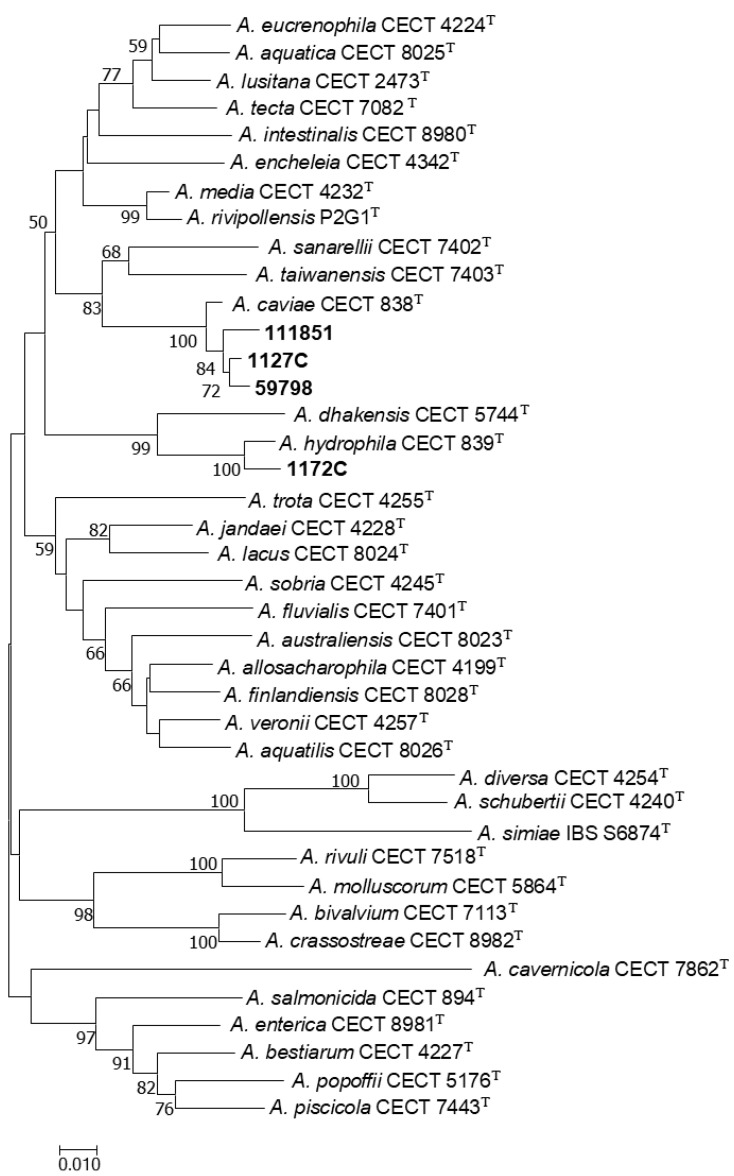
Phylogenetic tree based on the *rpoD* gene (451 bp) with a neighbor-joining (NJ) algorithm, showing the relationships of the four clinical *Aeromonas* strains used in this study and the 36 type strains of all *Aeromonas* spp. Numbers at nodes indicate bootstrap values (percentage of 1000 replicates). Bar 0.01 estimated nucleotide substitutions per site.

The alignment of the *rpoD* sequences of the strains 1127C, 1172C, 111851, and 59798 with *rpoD* sequences of the type strains of all *Aeromonas* spp. constructed with the ClustalW algorithm had a total length of 451 bp. The phylogenetic tree derived from this alignment, elaborated with the neighbor-joining (NJ) algorithm, is shown in Figure 1 and revealed that *rpoD* sequences of strains 1127C, 111851, and 59798 clustered with the sequence of *Aeromonas caviae* type strain CECT 838^T^, whereas the sequence of strain 1172C clustered with the type strain of *Aeromonas hydrophila* CECT 839^T^, thus precisely identifying these strains at the species level.

### 3.2. Virulence Gene Detection

The presence of virulence-associated genes in the four *Aeromonas* strains is summarized in Table 3. The most frequent virulence gene detected was *alt* (100%), followed by *ascF-G*, *flaA*, *act*, and *ast* (75%); and *ascV*, *aerA*, and *hlyA* (50%). In contrast, the *aexT* gene was not detected in any of the four strains.

### 3.3. Fenofibrate In Vitro Antimicrobial Activity

**Figure 2:**
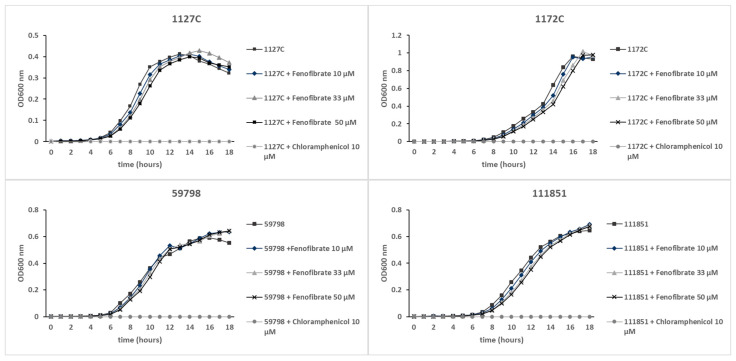
Growth curves of the *Aeromonas* strains 1127C, 1172C, 111851, and 59798 incubated with fenofibrate at 10 µM, 33 µM, and 50 µM, and without the drug. Positive control of antimicrobial activity was represented by bacteria incubated with chloramphenicol at 10 µM. Bacterial growth was measured by monitoring the OD_600_ nm of bacterial cultures in a 96-well microplate. Results are means of quadruplicate wells.

The bacterial growth curves of the four *Aeromonas* strains incubated with fenofibrate at the three concentrations tested (10, 33, and 50 µM) showed no differences from the growth of bacteria incubated without the drug, as can be observed in Figure 2. In contrast, chloramphenicol at 10 µM completely prevented the growth of bacteria. These results indicate that fenofibrate did not exhibit antimicrobial activity at these concentrations against the *Aeromonas* strains tested.

### 3.4. Fenofibrate Reduces the Intracellular Survival of Bacteria within Macrophages

**Figure 3:**
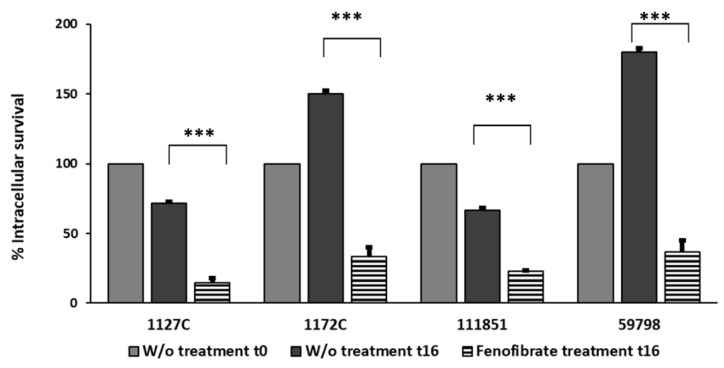
Effect of fenofibrate in the reduction in the percentages of intracellular survival of the four *Aeromonas* strains at MOI 10 in J774A.1 macrophages. For each strain, bacteria present in macrophages at t0 (light grey bars) were used as a reference value of 100% of initial bacterial burden. Values of CFU/mL within macrophages W/o treatment at t16 (dark grey bars) and fenofibrate-treated macrophages at t16 (striped bars) were used to calculate percentages of intracellular bacteria with respect to the CFU/mL in macrophages at t0. Significant differences *p* < 0.001 (***). Results are means ± SD from three independent experiments with three replicates in each experiment. W/o, macrophages without treatment.

The effect of fenofibrate in the clearance of intracellular bacteria present in macrophages is shown in Figure 3. In macrophages without treatment at t16, intracellular survival percentages were variable between strains; there were two strains in which these percentages decreased with respect to t0 (71.42% for strain 1127C and 66.66% for strain 111851) and two strains in which intracellular survival percentages increased (150% for strain 1172C and 180% for strain 59798), which means that the number of bacteria of these two strains increased inside the macrophages after 16 h. Regarding the survival percentages of bacteria in fenofibrate-treated macrophages at t16, significant reductions in all the strains (*p* < 0.001) were observed, with percentages of survival of 14.4% for 1127C, 33.3% for 1172C, 22.91% for 111851, and 36.5% for strain 59798.

### 3.5. Fenofibrate Decreases the Cell Damage Produced by Bacteria in Macrophages

**Figure 4:**
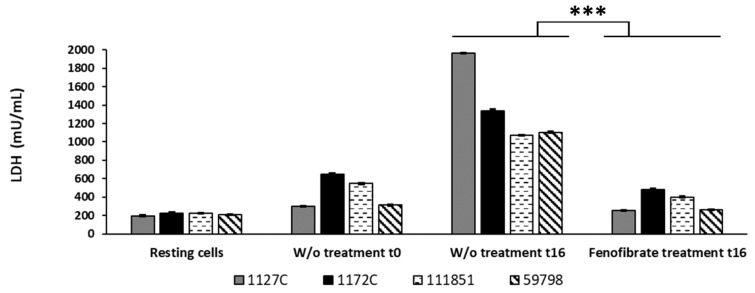
Effect of fenofibrate in the reduction in the cell damage in J774A.1 macrophages caused by the four *Aeromonas* strains at MOI 10. Cell damage was evaluated by measuring the release of lactate dehydrogenase (LDH) enzyme. LDH values of fenofibrate-treated macrophages at t16 were used to calculate percentages of cell damage reduction with respect to macrophages without treatment at t16. Significant differences *p* < 0.001 (***). Results are means ± SD from three independent experiments with three replicates in each experiment. W/o, macrophages without treatment.

Cell damage produced by the four *Aeromonas* strains in macrophages, measured as the release of LDH enzyme to the cell culture supernatants, is shown in Figure 4. In those macrophages without treatment, the four *Aeromonas* strains were able to produce time-dependent cell damage at MOI 10, the amount of LDH released at t16 being significantly higher than that produced at t0 (*p* < 0.001). 

Meanwhile, a significant decrease in the LDH released in the fenofibrate-treated macrophages compared to macrophages without treatment at t16 was observed for all the strains (*p* < 0.001). The percentages of reduction in cell damage of fenofibrate-treated macrophages at t16 compared to macrophages without treatment at t16 were 86.976%, 64.201%, 76.240%, and 62.713% for strains 1127C, 1172C, 59798, and 111851, respectively. 

### 3.6. Fenofibrate Has a Dose-Response Effect on the Viability of Macrophages Infected with Aeromonas caviae Strain 1127C

**Figure 5:**
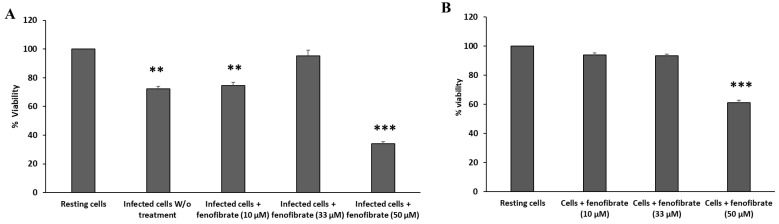
Fenofibrate dose–response effect on viability of J774A.1 macrophages: (**A**) infected with *Aeromonas caviae* strain 1127C at MOI 10 and (**B**) in the absence of infection. Macrophage viability was measured with MTT reduction assay after 12 h of incubation. Significant differences with respect to resting cells *p* < 0.01 (**) and *p* < 0.001 (***). Results are means ± SD from three independent experiments with three replicates in each experiment. W/o, macrophages without treatment.

The results of viability of infected macrophages incubated with different doses of fenofibrate, measured as the reduction of MTT, are shown in Figure 5. Infected macrophages without treatment showed a significant reduction in their viability with respect to resting cells (72.43%, *p*-value < 0.01). In the infected fenofibrate-treated macrophages, different percentages of viability for the three concentrations tested were observed. While macrophages incubated with fenofibrate at 10 µM showed a viability of 74.65%, similar to the viability observed in the infected macrophages without treatment, those incubated with 50 µM of fenofibrate showed a viability of 34.15%, a viability percentage even lower than that of infected macrophages without fenofibrate. The highest level of viability was observed for macrophages incubated with fenofibrate 33 µM (95.14% of viability), having a similar percentage of viability to resting cells (*p*-value < 0.01). Finally, macrophages incubated with fenofibrate 10 µM, 33 µM, and 50 µM in the absence of infection showed viability percentages of 94%, 93.4%, and 61.24% (Figure 5). This last percentage of viability was significantly lower than the viability of resting cells, indicating a potentially cytotoxic effect for macrophages.

### 3.7. Expression of PPAR-α and Immune-Related Genes in Macrophages Facing Aeromonas Infection Is Modulated by Fenofibrate

**Figure 6:**
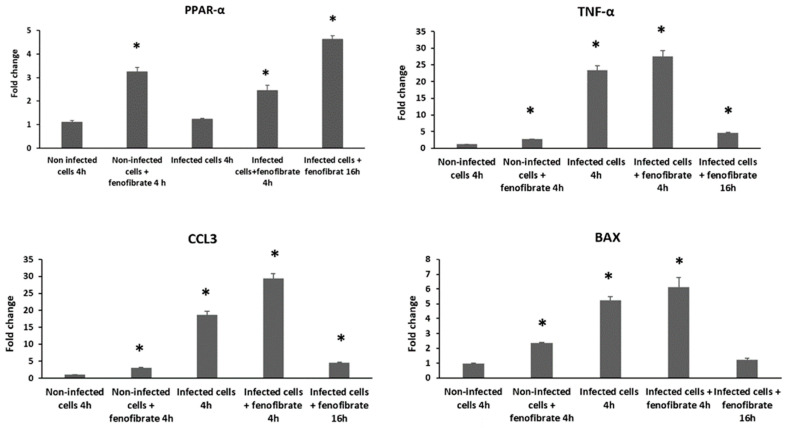
Gene expression profile determined by RT-qPCR of the *PPAR-α* gene and the immune-related genes *TNF-α*, *CCL3*, and *BAX* in J774A.1 macrophages infected with *Aeromonas caviae* strain 1127C at MOI 10 and fenofibrate treatment. Transcript levels of the genes were normalized to the expression of the *GAPDH* gene. Expression fold change with respect to the non-infected cells was calculated using the comparative ΔΔCt method. * Significant differences compared with non-infected cells *p*-value < 0.05. Results are means ± SD from three independent experiments with three replicates in each experiment.

The results of expression levels of the *PPAR-α* gene showed a direct time-dependent expression in those macrophages incubated with fenofibrate, as can be observed in Figure 6. At 4 h of incubation, non-infected and infected macrophages with strain 1127C, both incubated with fenofibrate, showed significant expression levels of *PPAR-α* in relation to resting cells (*p* < 0.05), while infected macrophages incubated in the absence of fenofibrate did not show this overexpression. Meanwhile, the expression of *PPAR-α* in infected macrophages incubated with fenofibrate for 16 h was significantly higher than the expression observed at 4 h for macrophages subjected to the same condition, i.e., infection and fenofibrate incubation (*p* < 0.05). Regarding the expression of the immune-related genes *TNF-α*, *CCL3*, and *BAX*, it was observed that at 4 h of incubation, both fenofibrate and strain 1127C separately induced a significant expression of these genes in relation to resting cells (*p* < 0.05) (Figure 6). This expression was even higher in macrophages when the two conditions, infection and fenofibrate incubation, were applied together. At 16 h of incubation, infected fenofibrate-treated macrophages showed lower expression levels of the three genes with respect to macrophages with the same condition at 4 h, reaching expression values similar to those of resting cells (*p* < 0.05).

### 3.8. Fenofibrate Reduces the Cell Damage and the Intracellular Survival after Pseudomonas aeruginosa Infection

**Figure 7:**
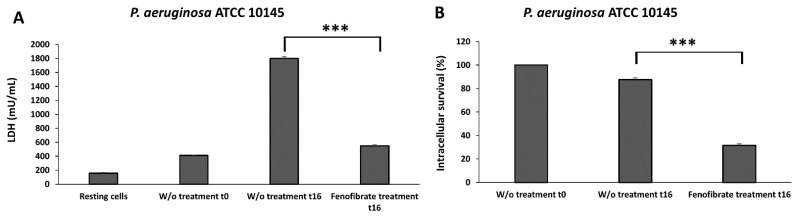
Effect of fenofibrate in the reduction of the cell damage (**A**) and intracellular survival (**B**) in J774A.1 macrophages caused by *Pseudomonas aeruginosa* strain ATCC 10145 at MOI 5. Significant differences *p* < 0.001 (***). Results are means ± SD from three independent experiments with three replicates in each experiment. W/o, macrophages without treatment.

Cell damage produced by the *P. aeruginosa* ATCC 10145 in macrophages, measured as the release of LDH enzyme to the cell culture supernatants, is shown in Figure 7A. In those macrophages without treatment, this strain was able to produce time-dependent cell damage at MOI 5 (*p* < 0.001). Meanwhile, a significant decrease in the LDH released in the fenofibrate-treated macrophages in relation to macrophages without treatment was observed at t16. The percentage of reduction in cell damage of fenofibrate-treated macrophages at t16 respect macrophages without treatment at t16 was 30.95%. The effect of fenofibrate on intracellular survival is shown in Figure 7B. In macrophages without treatment at t16, the intracellular survival percentage was less than at t0. The survival percentage of bacteria in fenofibrate-treated macrophages at t16, showed a significant reduction (*p* < 0.001), with a survival percentage of 31.52%.

## 4. Discussion

Fibrates have been considered as potential targets for repositioning, considering their positive effects both with in vivo and in vitro models subjected to bacterial challenge [44]. These compounds, derived from fibric acid, act as specific ligands to peroxisome-activated receptors (PPAR), a family of nuclear receptors from the nuclear hormone receptor superfamily that acts as transcriptional factors involved in different metabolic routes related mainly to energy homeostasis [20,21,45]. Fenofibrate is a third-generation fibric acid derivative that, once administered, is metabolized to fenofibric acid by tissue and plasma esterases [46]. Fenofibric acid binds to PPAR-α, located in the nuclear membranes of cells mainly in oxidative tissues like the liver, heart, skeletal muscles, brown adipose tissue, intestines, and kidneys, promoting a conformational change in the receptor structure and its subsequent action as a transcriptional factor [45]. Lipid metabolic processes involving PPAR-α transcriptional activity have been extensively studied, and include fatty acid transport, esterification, and oxidation. In addition, anti-inflammatory properties associated with PPAR-α activation have awakened a strong interest due to their therapeutic potential in diseases as diverse as vascular complications, diabetes, and liver disorders [22].

The interesting anti-inflammatory response mediated by the binomial PPAR-α and fenofibrate has been also studied as a way to diminish the harmful effects related to pathogen invasion and proliferation [47]. In this sense, this study aimed to assess the role of fenofibrate in improving the response of the murine macrophage cell line J774A.1 against infections caused by clinical *Aeromonas* strains, an emergent pathogen whose virulence mechanisms and interaction with eukaryotic hosts has been extensively studied and for which AMR phenomena has increased in recent decades [23,25]. The study first examined the potential antimicrobial activity of three concentrations of fenofibrate (10, 33, and 50 µM) later used in the following assays against four *Aeromonas* strains isolated from human stool samples with different antimicrobial susceptibility profiles and variability in the presence of virulence genes. The strains were identified by *rpoD* sequencing as *Aeromonas caviae* and *Aeromonas hydrophila*, two of the most common species found in clinical settings [25]. At the concentrations tested, fenofibrate did not show any antimicrobial effect on these strains, neither bactericidal nor bacteriostatic. In fact, the previously reported beneficial effects of fenofibrate in the course of bacterial infections are related to the activation of PPAR-α coupled with a reduction in inflammatory processes associated with pathogen virulence, rather than as a bactericidal or bacteriostatic drug [22]. For that reason, the protective role of fenofibrate was studied in an experimental infection model using the murine macrophage cell line J774A.1, and this role was first assessed by quantifying the intracellular survival of bacteria and the cell damage caused to the macrophages. For these experiments, fenofibrate was prepared at a concentration of 33 µM based on previous reports using this concentration in similar assays [17]. As has been demonstrated before, and confirmed with the strains used in this study, *Aeromonas* is an intracellular pathogen able to produce cell damage in different eukaryotic cell lines [48,49,50,51]. It was observed that macrophages subjected to fenofibrate treatment showed a reduction in the intracellular survival of the invading bacteria, thereby enhancing the process of bacterial clearance. This decrease in the bacterial load could be linked to the lower cell damage observed in fenofibrate-treated macrophages, as there was a smaller number of bacteria able to produce the cytotoxicity observed in macrophages without treatment. Similar results in the decrease in cell damage and intracellular survival were observed in our study after infection with *P. aeruginosa* ATCC 10145. Furthermore, the results of the MTT assay highlighted that the viability of macrophages is significantly improved by incubation with fenofibrate 33 µM more than at the 10 and 50 µM doses, a result in concordance with that observed by Andersson et al., in which fenofibrate 33 µM increased the viability of RAW264.7 macrophages infected with the highly virulent strain CO92 of *Yersinia pestis* [17]. The decrease in the survival and cytotoxicity of intracellular pathogens related to fenofibrate administration have also been reported in some in vivo studies, in which fenofibrate-treated groups had higher survival rates when exposed to infections [16,18,52]. Other studies have also linked the use of specific ligands of PPAR-α and the reduction in intracellular pathogen burden and cytotoxicity in macrophages, as is the case of gemfibrozil for *Legionella pneumophila* and *Mycobacterium tuberculosis* [53], and palmitoylethanolamide for *Escherichia coli* [15].

Results of RT-qPCR showed that the expression of the *PPAR-α* gene was directly linked to fenofibrate incubation, agreeing with previous studies in which fenofibrate promotes an increase in the expression of this gene [18,54]. Meanwhile, the expression of innate immune-related genes *TNF-α*, *CCL3*, and *BAX* was upregulated both by infection and fenofibrate at an early stage of incubation (4 h), with higher expression levels with the merge of infection and fenofibrate than by the two conditions separately. However, the expression of these genes was downregulated at a longer exposure time of 16 h. Tumor Necrosis Factor-α (TNF-α) is an inflammatory cytokine generated by macrophages/monocytes in the course of acute inflammation. It plays a pivotal role in initiating various signaling events within cells, ultimately contributing to necrosis or apoptosis [55]. The chemokine CCL3 plays a role in directing immune system cells through chemotaxis, including monocytes/macrophages, and it also contributes to the regulation of intracellular signaling mechanisms involved in inflammation [33]. Finally, the *BAX* gene encodes for the Bcl-2-associated X protein (BAX), which has a critical role in mitochondrially regulated cell death by permeabilizing the outer mitochondrial membrane. Because the physiological role of BAX is to maintain cell and tissue homeostasis, any dysregulation of BAX results in abnormal cell death [56]. It has been previously reported that some species of *Aeromonas* are able to induce the overexpression of proinflammatory cytokines, chemokines, and genes related to apoptosis [33,57], as we have observed with the strains studied in this work. In some cases, this overexpression is maintained during infection and is partially responsible for cytokine storm, a systemic inflammatory response that increases the infection severity [58]. Additionally, as indicated above, we observed that fenofibrate promoted an upregulation of the three genes at an early stage of incubation (4 h), less evident in the case of *TNF-α* and *CCL3*, and more marked in the case of the *BAX* gene. In this last sense, it has been observed in some studies that fenofibrate could promote the expression of pro-apoptotic signaling molecules and effectors. For example, Binello et al. determined that fenofibrate induced pro-apoptotic and anti-proliferative effects in high-grade glioma (HGG) cells [59]. Our results indicate that the upregulation of the expression of innate-immune genes, induced by bacteria and enhanced with fenofibrate incubation, could be responsible for the elimination of the internalized bacteria by macrophages at the first term. Then, the upregulated expression of *PPAR-α* mediated by fenofibrate reduces the overexpression of the innate-immune genes and an exaggerated inflammatory response is ameliorated. In agreement with these results, previous studies have related the upregulated expression of *PPAR-α* by fenofibrate with the reduction in the expression of genes encoding for proinflammatory cytokines and chemokines, including *TNF-α* and *CCL3*, and apoptosis genes like *BAX* [18,60,61]. The expression of *PPAR-α* induced by specific ligands, as in the case of fenofibrate, has been observed to have implications in the reduction of various inflammatory parameters, such as inhibition of the expression of tissue factor, cyclooxygenases, or other proinflammatory mediators [62,63,64]. 

## 5. Conclusions

Fenofibrate, a drug generally used in the treatment of hyperlipidemic disorders, has been shown to have potential protective effects in macrophages facing *Aeromonas* infection and infections by other pathogens such as *Pseudomonas aeruginosa*. Fenofibrate improves the response of macrophages against infection by reducing both macrophage death and the clearance of internalized bacteria and increasing the viability of macrophages with a dose-response effect. Fenofibrate modulation of the expression of genes related to innate immunity *TNF-α*, *CCL3*, and *BAX*, as well as the gene encoding for its cell receptor PPAR-α, could be part of the pathways by which bacterial cells are eliminated, and therefore this protection is achieved. These findings suggest that fenofibrate may have beneficial effects in the treatment of bacterial infections, which could represent a successful strategy of drug repositioning. Nevertheless, further research and clinical evaluations are needed to fully understand the potential therapeutic role of fenofibrate against infections and its optimal use in the clinical setting.

## Figures and Tables

**Table 1:** Antimicrobial susceptibility profile *of Aeromonas* strains used in this study.

Antimicrobial	1127C	59798	1172C	111851
Cefepime	R	S	S	S
Cefotaxime	R	S	R	S
Ceftazidime	R	S	I	S
Meropenem	R	S	S	S
Ertapenem	R	S	S	S
Imipenem	R	S	R	S
Aztreonam	S	S	S	S
Piperacillin-tazobactam	R	S	R	R
Ciprofloxacin	S	S	S	S
Levofloxacin	S	S	R	S
Cotrimoxazole	R	S	S	R
Tetracycline	S	S	R	S
Gentamicin	S	S	S	S
Amikacin	S	S	S	S

**Table 3:** Presence of virulence genes of *Aeromonas* strains used in this study.

Strain	*ascF-G*	*ascV*	*aerA*	*hlyA*	*flaA*	*act*	*ast*	*alt*	*aexT*
1127C	+	+	+	−	+	+	+	+	−
1172C	+	−	−	+	+	−	+	+	−
59798	−	−	−	−	+	+	+	+	−
111851	+	+	+	+	−	+	−	+	−

Notes: +, detected; −, not detected.

## Data Availability

Data are contained within the article.
